# Detection of *Plasmodium falciparum-*infected *Anopheles sergentii* with reduced insecticide net susceptibility in a dryland ecosystem of the Kenyan Rift Valley: implications for malaria surveillance and vector control

**DOI:** 10.1186/s13071-026-07601-z

**Published:** 2026-08-09

**Authors:** David P. Tchouassi, Gilbert Rotich, Amine M. Mustapha, Fidel G. Otieno, Luna Kamau, Ambrose Oruni, Baldwyn Torto, Charles S. Wondji

**Affiliations:** 1 International Centre of Insect Physiology and Ecology, PO Box 30772, Nairobi, 00100 Kenya; 2 Department of Zoology and Entomology, University of Pretoria, Private Bag X323, Pretoria, 0001 South Africa; 3 Centre for Biotechnology Research and Development, Kenya Medical Research Institute, PO Box 54840, Nairobi, 00200 Kenya; 4 Centre for Research in Infectious Diseases, PO Box 13591, Yaoundé, Cameroon; 5 Vector Biology Department, Liverpool School of Tropical Medicine, Liverpool, L3 5QA UK

**Keywords:** Dryland ecosystems, Emerging malaria vectors, Malaria surveillance, Vectorial capacity, Cryptic vectors, Outdoor biting

## Abstract

**Background:**

In Kenya, parts of the dryland ecosystems have disproportionately witnessed a surge in malaria burden amidst poor understanding of the entomologic risk factors. This study investigated whether this trend is linked to shifts in the vector landscape, including the cryptic role of potential new vectors.

**Methods:**

Adult female anopheline mosquitoes were collected in a village in semi-arid to arid Marigat subcounty, Baringo County of the Kenyan Rift Valley. This was achieved using paired CDC light traps placed indoors and outdoors in randomly selected households during sampling periods, in August 2023, August 2024, and July 2025. A subset of the mosquitoes morphologically identified as *Anopheles funestus* (s.l.) were analysed by PCR and sequencing for species identification, malaria parasite infection and host meal sources. In addition, using cone bioassays, the efficacy of single and dual-ingredient insecticide-treated nets was evaluated on the mosquito population.

**Results:**

Analysis of the mosquitoes revealed presence of established *Anopheles sergentii* populations infected with *Plasmodium falciparum* (overall infection rate 8.9%, 27/302) in 2023, 2024, and 2025. *An. sergentii* exhibited low anthropophagy (~ 8%) but high plant-feeding rates (~ 85%). Despite its predominant outdoor activity evidenced from high representation among infected mosquitoes outdoors (25/290) than indoors (0/12), mortality remained low (8–53%) after exposure to piperonyl butoxide- and pyriproxyfen-treated bed nets. Five *An. longipalpis* C specimens were also found infected with *P. falciparum* (1.7%; 5/302).

**Conclusions:**

We provide evidence of *An. sergentii*, beyond its historical record in Kenya, as a potential malaria vector, underscoring the need to mainstream molecular analysis into routine malaria surveillance activities. The reduced efficacy of dual-ingredient nets against *An. sergentii* could present a serious challenge to their future deployment in the region, including outdoor control tools with similar active ingredients. This mosquito, and cryptic malaria vectors, such as *An. longipalpis* C could pose additional threat to malaria control efforts in Kenya.

**Graphical Abstract:**

**Figure:**
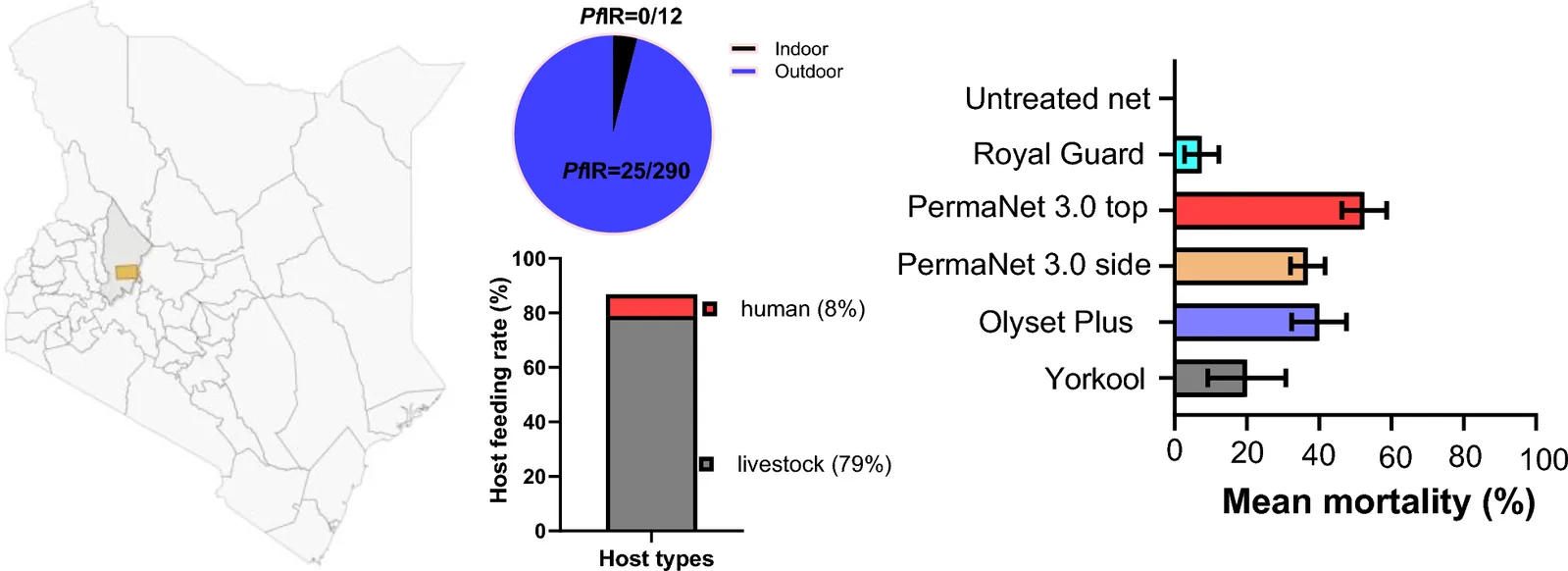

**Supplementary Information:**

The online version contains supplementary material available at https://doi.org/10.1186/s13071-026-07601-z.

## Background

Mosquito-borne diseases remain a major cause of morbidity and mortality across the tropical regions, globally. For instance, despite much progress in the control of malaria, malaria-associated morbidity remains high mostly in sub-Saharan Africa [1]. Among the biological threats to effective control of the disease include the evolution and spread of insecticide resistance and the spread of efficient invasive malaria vectors such as *Anopheles stephensi* and *An. albimanus* [2]. Thus, malaria surveillance remains critical in reducing the burden of the disease which is central to the global technical strategy [3].

The Kenyan malaria control program achieved a substantial reduction in malaria incidence and mortality between 2015 and 2020. However, between 2019 and 2023, shifts occurred in the epidemiological landscape, with marked geographic variations in malaria incidence. Notably, malaria incidence has doubled in regions with drier, arid to semi-arid ecologies that were previously considered seasonal, low-transmission areas [4]. This increase in incidence coincides with changes in the malaria transmission profile, including the introduction of the invasive *An. stephensi* [5], the detection of *An. coluzzii*, previously thought to be restricted to West and Central Africa [6], and, unexpectedly, the endemic prevalence of *Plasmodium vivax* in Duffy-negative individuals [7]. We hypothesize that changes in the malaria vector landscape will exacerbate residual transmission in the more arid ecosystems of Kenya. To date, few field studies have focused on the entomological drivers of malaria in Baringo County, which is among Kenya’s ASAL (arid and semi-arid lands) counties that were recently reclassified as moderate-to-high endemic areas [4].

In this study, we aim to explore poorly understood entomological drivers sustaining malaria transmission in the dryland ecosystem of Kenya. Here, we report evidence of *Anopheles sergentii* beyond its historical record in Kenya [8] as a malaria vector exhibiting high *Plasmodium* *falciparum* infection rate but zoophagic tendency, high dependence on plant feeding for survival, and demonstrating reduced sensitivity to single and dual-ingredient insecticide-treated bed nets despite predominant outdoor biting activities. These findings contribute valuable evidence on the determinants of residual malaria transmission and support the implementation of appropriate malaria control interventions.

## Methods

### Study site and mosquito collection

**Fig. 1:**
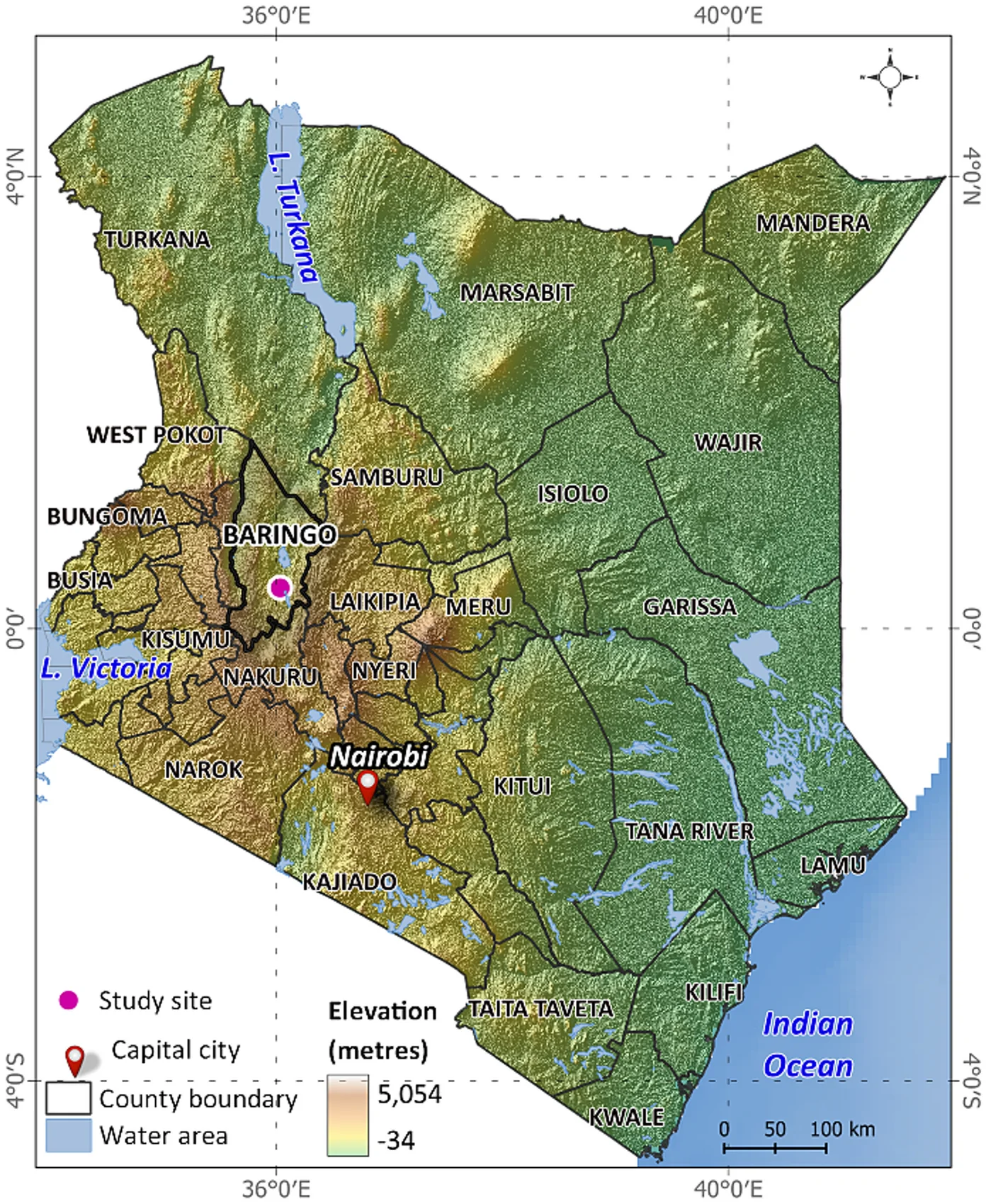
Study area within Marigat subcounty, Baringo County, Kenya

Marigat subcounty where mosquitoes were collected (Fig. 1) lies in the lowland zone of Baringo County (900–1000 m asl) and malaria transmission is highly seasonal peaking during the rainy season [9]. Daily temperatures range 3–37 °C with annual rainfall averaging 650 mm. The inhabitants mostly engage in agro-pastoral agriculture as a source of livelihood.

Adult female mosquitoes were sampled using CDC light traps (Model 512, John W. Hock Company, Gainesville, FL, USA). Collection was achieved using paired traps set indoors and outdoors (19:00–6:00 h) in at most 10 randomly selected households during sampling periods, in August 2023, August 2024, and July 2025, corresponding to the periods after the rains.

### DNA extraction, amplification and sequencing

A subset of mosquitoes initially identified morphologically as *Anopheles funestus* s.l., was randomly selected and processed for molecular analysis guided by important considerations including trophic status, indoor versus outdoor location and available resources.

Genomic DNA (gDNA) was isolated from head/thorax and abdominal regions of individual mosquitoes using the ammonium acetate protein precipitation method [10]. The gDNA from head/thorax region was processed to screen for *Plasmodium* infection (sporozoites) and mosquito species identification. The DNA from abdominal portion was analysed for sources of vertebrate host blood as well as presence of host plant DNA and species.

*Plasmodium* infection was detected by real-time PCR assay of the cytochrome b (*cytb*) gene [11]. Subsequently, the protocol was adapted in a conventional PCR and portion of the *cytb* amplicons (~ 367 bp) from a subset of samples Sanger sequenced. To confirm presence of sporozoites, segments of approximately 319 nucleotides of the *P. falciparum* circumsporozoite (csp) gene were amplified by PCR [12] and sequenced from both strands of each purified PCR product, outsourced to Macrogen Europe BV (Amsterdam, The Netherlands).

To confirm the mosquito host identity, ribosomal second internal transcribed spacer (ITS-2) [13] and the mitochondrial cytochrome c oxidase I (*cox-1*) [14] loci were amplified via PCR assays from all *Plasmodium-*positive samples and 10 randomly selected *Plasmodium-*negative individuals and sequenced. The analysis followed established protocols [15].

Blood meal analysis was performed using high-resolution melt-PCR of the *cytb* gene [15] on abdominal genomic DNA from both *Plasmodium*-infected and select uninfected mosquitoes*.* Host blood meal identification was achieved by comparing HRM melt curves to those of authentic standard of reference vertebrate control species (cow, sheep, goat, chicken, swiss mouse, monkey, bird and human).

Finally, to detect evidence of plant feeding using PCR, the chloroplast ribulose-1,5 bisphosphate carboxylase gene was amplified in abdominal gDNA of *Plasmodium*-infected mosquitoes and sequenced as previously described [16].

Primers used in this study are included in Table S1. Negative controls containing nuclease-free water were included in all PCR runs.

### Sequence analysis

Sequences for each target were edited and aligned using ClustalW or MUSCLE in MEGA v7 [17]. With default parameters and trimmed to a uniform length corresponding to the amplified fragment. Sequences were confirmed by comparison against the NCBI GenBank database using BLAST nucleotide function. Phylogenetic reconstruction for each of the gene sequences, with the best-fit model of DNA sequence evolution implemented in MEGA v7, and nodal support evaluated by 1000 bootstrap resampling. Additionally, for *cox-1* mosquito sequences, the percent evolutionary divergence between the species and reference ones in the NCBI GenBank was estimated.

### Insecticide-treated bed net efficacy assessment

During the 2025 collection, five live blood-fed mosquitoes (obtained from 4 individual traps in separate households) were maintained (~ 3 days) until they were gravid, from which eggs obtained using the forced egg-laying technique were reared to adults under standard conditions [18]. Following WHO guidelines, cone bioassays [19] were conducted to evaluate the efficacy of several single and dual-ingredient insecticide-treated nets (Table 1). In the assay, non-blood-fed, 2- to 5-day old F1 female *Anopheles sergentii* were placed in plastic cones enclosed with the mosquito net during 3 min of exposure. Control (untreated) nets were included, and mortality was assessed 24 h post-exposure while mosquitoes were maintained on 10% sugar solution provided on soaked cotton. Three to eight replicates of 5–10 F1 mosquitoes were tested per net type totaling 261 mosquitoes. Except for Yorkool, net compositions and manufacturers are described in Oruni et al. [20]. Yorkool net is distributed by the Kenyan National Malaria Control program largely in coast and western regions.

**Table 1:** List of bed nets tested in cone assays and associated characteristics

Bed net type	Description	Manufacturer
Yorkool	Deltamethrin 2% 55 mg/m^2^ impregnated on all sides	Yorkool chemicals Co, Ltd, Cangzhou, China),
Olyset plus	800 mg/m^2^ of Permethrin + 400 mg/m^2^ PBO impregnated in polyethylene material	Sumitomo chemical
Permanet 3.0 only side	84 mg/ m^2^ of deltamethrin on the sides and 120 mg/m^2^ on the top + 800 mg/m^2^ PBO on the top impregnated in polyester material	Vestergaard
Permanet 3.0 only top		
Royal guard	208 mg/m^2^ of alpha-cypermethrin + 208 mg/m^2^ of Pyriproxyfen impregnated in polyethylene material	Disease control technologies (DCT)
Untreated (control)	100% polyester with no insecticide	Local market

### Statistical analysis

The Chi-square goodness-of-fit test was used to compare proportion of mosquitoes indoors and outdoors. The human blood index was expressed as the proportion of blood-feeding on humans of the total number of blood-fed mosquitoes examined. All analysis was performed in R v 4.5.1 (The R Foundation for Statistical Computing) and results considered significant at *p* ≤ 0.05. Mortality rate was calculated as a percentage of dead mosquitoes of the total exposed and confidence intervals estimated using the binom package in R 4.5.1. The effect of net type on mosquito mortality was tested using binomial regression model. In addition, pairwise test of proportion of mosquito mortality between the nets was compared using Pearson’s Chi-square test with Bonferroni correction.

## Results and discussion

### Mosquito abundance and *Plasmodium* infection rate

**Fig. 2:**
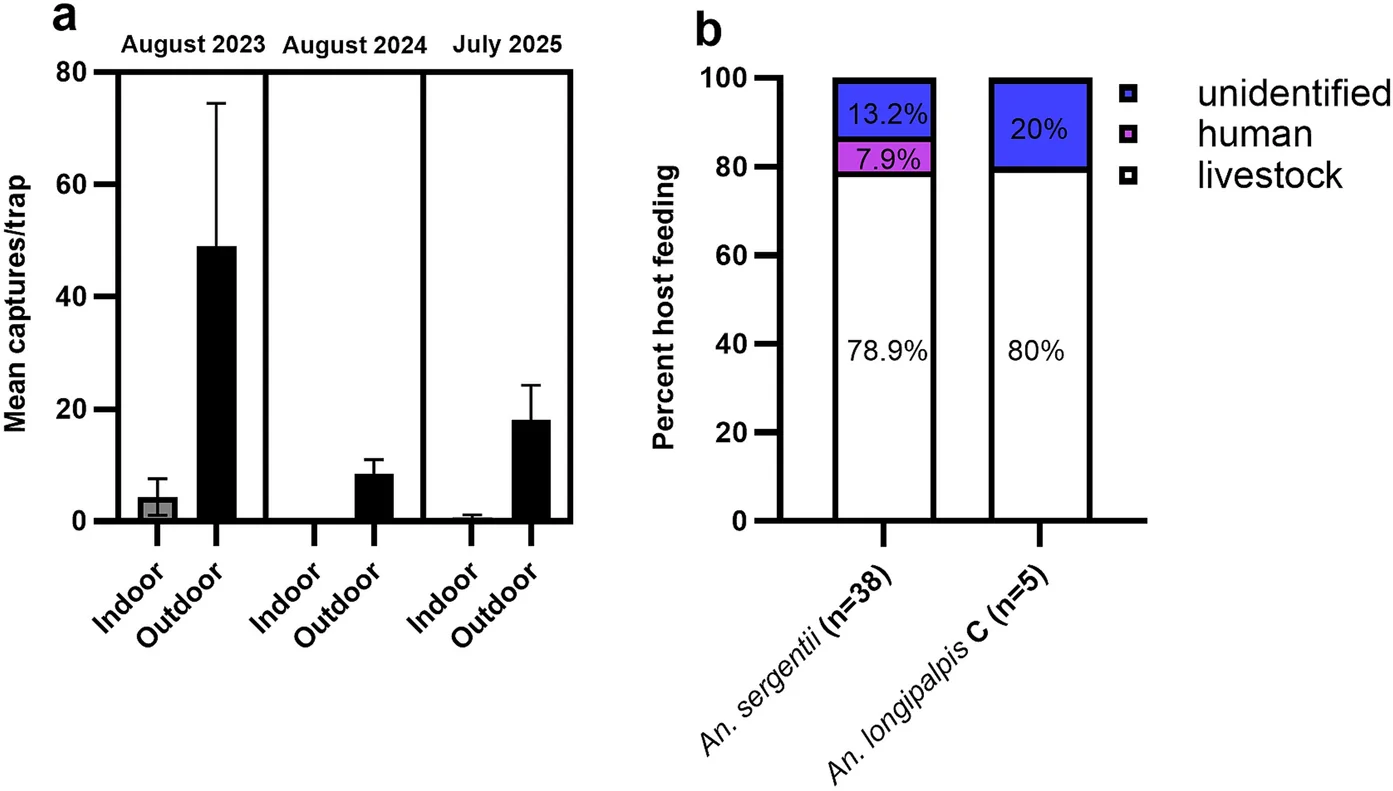
Bionomics of *Anopheles sergentii* detected in Marigat subcounty, Baringo County, Kenya. **a** *Anopheles. funestus* s.l. captures, **b** vertebrate host meal pattern of *An. sergentii* and *An. longipalpis* C. Error bars represent the standard error of the mean; n, number of samples analysed for each species. The number of samples analysed were 153, 17 and 132 in August 2023, August 2024 and July 2025, respectively

Significantly more of the *An. funestus* s.l. mosquitoes were collected outdoors than indoors (*n* = 629; χ^2^ = 463.59, df = 1, *p* < 0.0001) across the randomly selected households in August 2023, August 2024, and July 2025 (Fig. 2a).

gDNA extract was obtained from a subset of individual mosquitoes (48%, 302/629; 214 blood-fed, 88 unfed). Of these, 32 female *Anopheles* mosquitoes tested positive for *P. falciparum* sporozoites with an overall infection rate of 10.6% (32/302), and these samples were exclusively associated with outdoor collections: (2023: 8.5% (13/153); 2024: 23.5% (4/17); 2025: 11.4% (15/132). A subset of *cytb* amplicons was Sanger sequenced (~ 367 bp; accession numbers PZ176374-85) which was 100% identical to *P. falciparum* isolates in NCBI GenBank. In addition, a fragment of the *P. falciparum* circumsporozoite gene (CSP, 319 bp; accession numbers PZ176370-72) was amplified and sequenced from three of the *P. falciparum-*positive mosquitoes, further confirming sporozoite infection.

### Molecular identity of mosquitoes

**Fig. 3:**
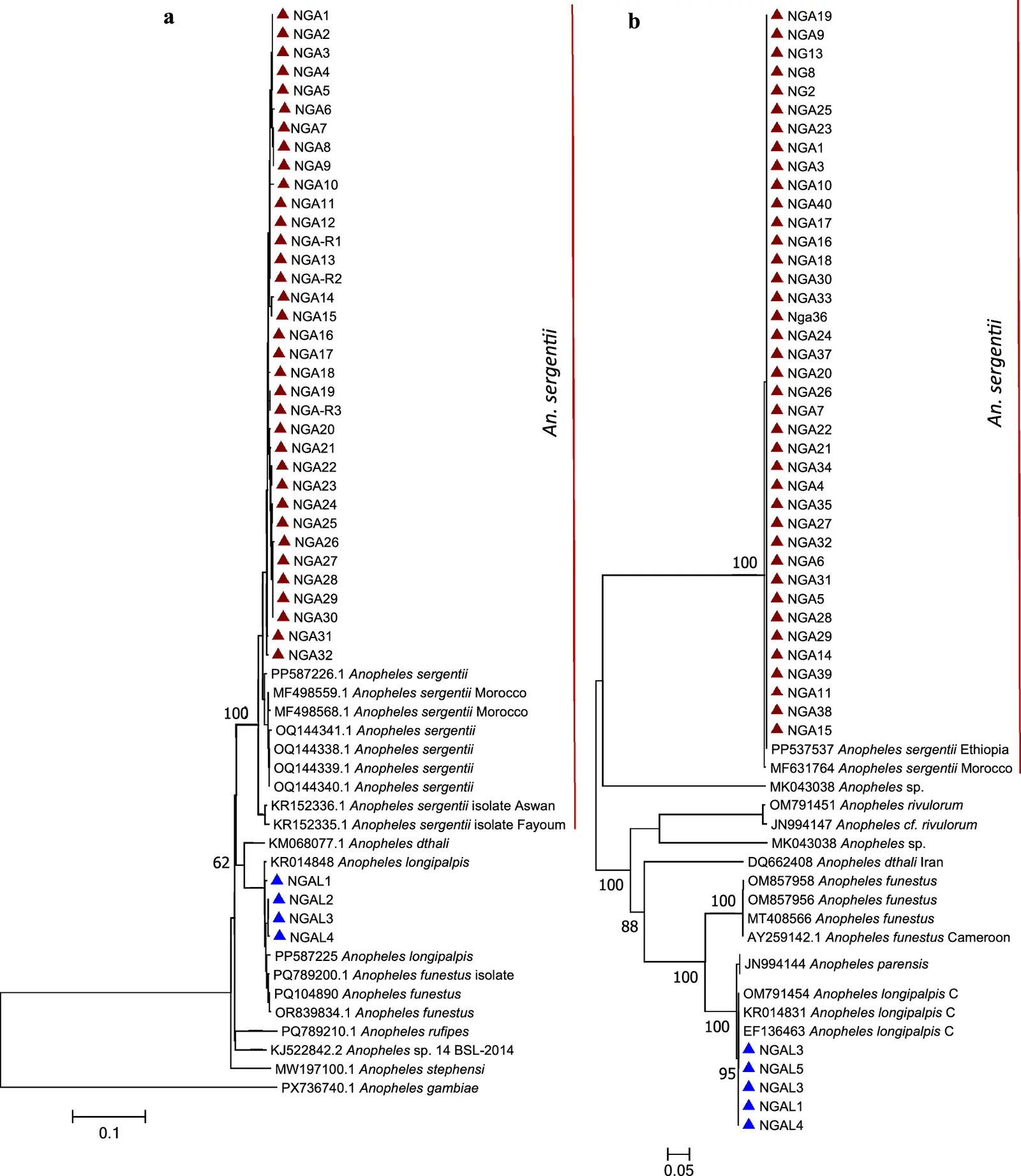
Evolutionary relationship of *An. sergentii* detected in Kenya inferred using neighbour-joining method. **a** *cox-1* sequences, **b** ITS-2 barcode region. Analysis was performed in MEGA v.7 with bootstrap support values based on 1000 replications shown above nodes. Other reference anopheline species are included for comparison. The scale-bar indicates the number of substitutions per site. Study samples for *Plasmodium* infected and non-infected *An. sergentii* specimens are indicated with red block triangles and infected *An. longipalpis* C in blue. Taxon abbreviations denote samples analysed and sequences deposited in NCBI GenBank for *cox-1* (*An. sergentii*: 542 bp, PZ164096-PZ164130; *An. longipalpis* C: 542 bp; PZ164131- PZ164134), ITS-2 (*An. sergentii*: 496 bp; PZ165444-82; *An. longipalpis* C: 252–261 bp; PZ165483-87)

To confirm the mosquito host identity, ITS-2 and *cox-1* sequences were generated from all *Plasmodium-*positive specimens (*n* = 32) and included randomly selected *Plasmodium-*negative individuals. DNA sequences from all positive samples corresponded to GenBank sequences of *An. sergentii* (84.4%, 27/32) and *An. longipalpis* C (15.6%; 5/32) (Fig. 3a, b), while all negative specimens (*n* = 10) were 100% identical to *An. sergentii* (Fig. 3a, b). Phylogenetic analysis showed that ITS-2 and *cox-*1 sequences clustered closely with reference *An. sergentii* from Ethiopia and Morocco. Kenyan *An. sergentii cox-1* sequences exhibited a mean evolutionary divergence of 1.9% from GenBank references indicative of intraspecific differences by region. These findings confirm the presence of *An. sergentii* and suggest that it is likely the predominant outdoor malaria vector among the specimens analysed. Its occurrence in this dryland ecosystem aligns with its known adaptation to extreme climates, known commonly as the ‘‘desert malaria vector’’, particularly in the Middle East and North Africa, including Morocco, Egypt, and Yemen [21]. Together, these results provide evidence of *An. sergentii*, beyond its historical record in Kenya [8], as a potential malaria vector.

### Host meal associations

Blood meal analysis from both *Plasmodium*-infected and uninfected *Anopheles sergentii* revealed low human feeding rates (7.9%; 3/38) and predominantly livestock feeding (78.9%; Fig. 2b) including goats (71.4.9%), sheep (14.3.1%) and domestic chicken (14.3%) as well as rodent (5.3%). Similarly, the few infected *An. longipalpis* C (*n* = 5) used non-humans as blood meal sources. Primarily feeding on animals can enhance the survival of the vector populations through the provision of blood meals thereby contributing to malaria persistence but opens opportunities for control using zoophylaxis. The low feeding is reminiscent of highly zoophilic mosquito vectors like *An. stephensi* with similar proclivity for arid ecologies [5, 22]. However, the low number of samples analysed may limit broad inference and interpretation of the data on blood meal hosts.

The high *Plasmodium* detection rate may seem incongruent with the low feeding rate on humans. However, human-feeding frequency may have been underestimated due to our PCR approach that may have been biased toward the most recent and abundant host DNA. Recent studies show high sensitivity of next generation sequencing approaches for host meal source identification including resolution of multiple host sources [23, 24] and this should be considered in the future.

Plant DNA was also detected in 85% (23/27) of *Plasmodium*-infected specimens based on amplification of the chloroplast ribulose-1,5 bisphosphate carboxylase gene. However, success rate of sequencing was low (6/23) revealing putative identified plant species based on BLASTn searches mostly corresponding to acacia (Fabaceae, *n* = 3), *Salvadora* sp. (Salvadoraceae, *n* = 1), *Citrus* spp. (Rutaceae, *n* = 2). These results indicate high plant-feeding rates, with sugar likely to play a significant role in survival and population dynamics as previously reported [25].

### Efficacy of insecticide-treated bed nets

**Fig. 4:**
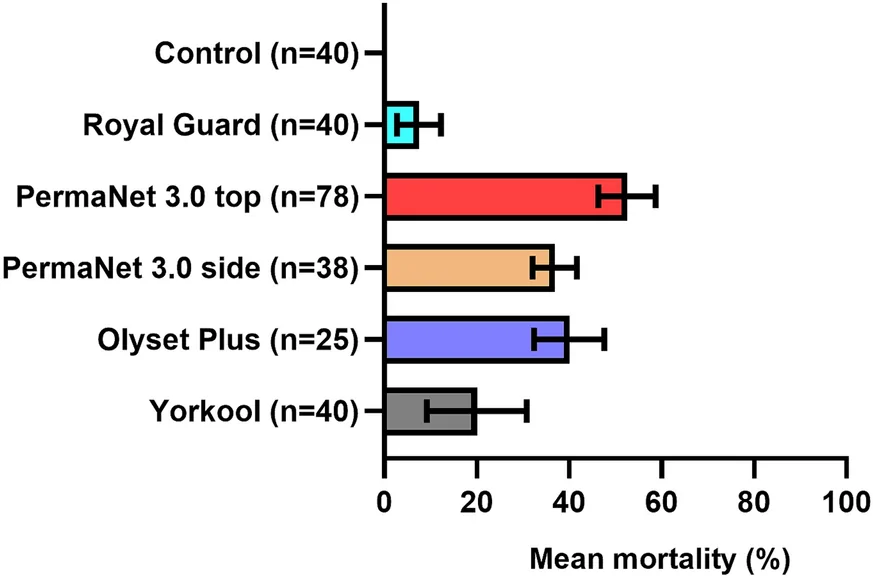
Bioefficacy of different commercial long-lasting insecticidal nets against *An. sergentii*; Error bars represent the standard error of the mean. Numbers in bracket indicate the number of mosquitoes evaluated for each net type or control

After 3-min exposure in cone assays, 24-h mortality of *An. sergentii* was lowest for the pyriproxyfen (PPF)-treated bed net Royal Guard (7.5%, CI 4.8–19.5%), followed by pyrethroid only Yorkool (20%, CI 10.5–34.8%) and PermaNet 3.0 side (36.8%, CI 23.4–52.7%). Moderate mortalities were induced by PBO-based nets (Olyset Plus = 40%, CI 23.4–59.2%; PermaNet 3.0 top: 52.5%, CI 41.6–63.3%). Mortality in untreated control nets was 0% (Fig. 4). Binomial regression model showed mosquito mortality varied by net type (χ^2^ (5, 21) = 24.57; *p* < 0. 0001). Further pairwise test of proportion of mosquito mortality between the nets, revealed significantly reduced performance of RoyalGuard compared to the other nets (*p* < 0.05). Mortality induced by PermaNet 3.0 top was significantly higher than that observed for Yorkool (*p* = 0.01).

These findings indicate reduced sensitivity of *An. sergentii* to both single- and dual-active ingredient bed nets, prior to their implementation in the study area. Notably, the reduced efficacy is comparable to levels observed for the major malaria vectors such as *An. gambiae* and *An. funestus* in other African countries [19, 26, 27], likely reflecting high levels of insecticide resistance in this mosquito population. This is particularly concerning, as these components, together with pyrethroids, are intended to enhance the killing of pyrethroid-resistant mosquitoes and improve the overall impact of ITNs.

The reduced net efficacy against *An. sergentii* could present a serious challenge to their future deployment in the region, including outdoor control tools with similar active ingredients. The study area is neither the focus of any malaria surveillance nor any insecticide-based intervention such as ITNs, indoor residual spraying or larval source management [4]. Thus, studies should investigate the source of potential resistance associated with this mosquito population.

This study has limitations. First, the geographic scope of the study focused on a single village; interest in analysing the mosquito samples was occasioned by anecdotal reports of increased malaria cases in the village. Although we found established populations across years, it remains unknown how widespread the species is in Kenya. There is clear need for investigations addressing bionomics, behaviour, ecology and genomic surveillance to elucidate the introduction and spread of this species in Kenya and across semi-arid regions of East Africa. Second, the largely outdoor activity of *An. sergentii* poses a challenge to colonizing the species in the laboratory. The few F1 derived from field collected gravid mosquitoes allowed us to test bed net efficacy providing only an indirect inference on resistance associated with this mosquito population based on reduced mortalities. Phenotypic assessment of insecticide resistance profiles is clearly needed. Third, our bed net efficacy was limited to cone assays which could grossly underestimate the killing effect of ingredients such as pyriproxyfen that requires wind tunnel or experimental huts to adequately assess their impact [20]. Fourth, the chemical content of the nets was not analysed to confirm the quantity of the active ingredients on the nets are those recommended by the manufacturers. One cannot rule out potential loss of active ingredients in the nets, and this should be investigated further. However, in the absence of a known mosquito susceptible strain in cone assays, we confirmed 100% mortality induced by Yorkool ITNs when exposed to F1 *An. parensis* (reared from gravid *An. funestus* s.l. from another site and related project). This finding indeed supports the loss of efficacy against the field-resistant *An. sergentii* populations. Fifth, based on our PCR-based method we uncover high *P. falciparum* rates analysing the head/thorax region and assume the infections were sporozoites. While the method aligns with recent studies [28, 29], incorporation of comparative markers and methodologies may improve reliability in the parasite detection profile [30]. Nonetheless, sequence data for the detected parasite (sporozoites) and focusing on the head/thorax region lend credence to maximizing chances of sporozoite detection.

## Conclusions

We provide evidence of *An. sergentii*, beyond its historical record in Kenya [8], as a potential malaria vector, underscoring the importance to mainstream molecular analysis into routine malaria surveillance activities. Much remains to determine its vectorial capacity and potential role in malaria transmission. If this is the case, this mosquito species may pose an additional threat to malaria control efforts in Eastern Africa that are already challenged by the invasive *An. stephensi* and cryptic malaria vector species such as *An. longipalpis* C identified in the current study and previously [31].

## Supplementary Information

Supplementary Material 1.

## Data Availability

The data supporting the conclusions of this article are included within the article and its supplementary files. All newly generated sequences were deposited in the NCBI GenBank database under the following accession numbers: PZ176374-8; PZ176370-72; PZ164096-PZ164130; PZ164131-PZ164134, 496 bp; PZ165444-82; PZ165483-87.

## Abbreviations

ASAL: Arid and semi-arid lands
asl: Above sea level
CDC: Centers for Disease Control and Prevention
csp: Circumsporozoite protein
*cox-1*: Cytochrome c oxidase subunit 1
*cytb*: Cytochrome b
DNA: Deoxyribonucleic acid
HRM: High-resolution melting
ITS-2: Second internal transcribed spacer
PCR: Polymerase chain reaction
s.l.: Sensu lato
