# Supplementary material for: Detection of Plasmodium falciparum-infected Anopheles sergentii with reduced insecticide net susceptibility in a dryland ecosystem of the Kenyan Rift Valley: implications for malaria surveillance and vector control
Source: Parasit Vectors. 2026 Aug 9;19:475. doi: 10.1186/s13071-026-07601-z (PMC13737560; doi:10.1186/s13071-026-07601-z)
Supplement: Supplementary file 1 [file 13071_2026_7601_MOESM1_ESM.docx]

| Target organism | Marker | Forward primer | Reverse primer | Reference |
| --- | --- | --- | --- | --- |
| *Plasmodium* spp. | cytb | TGGTAGCACAAATCCTTTAGGG | TGGTAATTGACATCCAATCC | Xu et al. 2015 |
| *Plasmodium* spp. | csp | TTAAGGAACAAGAAGGATAATACCA | AAATGACCCAAACCGAAATG | Lapp et al 2022 |
| Mosquito | ITS-2 | TGTGAACTGCAGGACACAT | TATGCTTAAATTCAGGGGGT | Beebe and Saul, 1995 |
| Mosquito | *cox-1* | GGTCAACAAATCATAAAGATATTGG | TAAACTTCAGGGTGACCAAAAAATCA | Folmer et al 1994 |
| Mosquito blood meal soources | cytb | CCCCTCAGAATGATATTTGTCCTCA | CATCCAACATCTCAGCATGATGAAA | (Ogola et al., 2019) |
| Plant meal source | rbcl | ATGTCACCACAAACAGAGACTAAAGC | GTAAAATCAAGTCCACCRCG | Kinya et al 2022 |
